# Reciprocal Hybridization Between Herbivorous and Carnivorous Sub-Cold-Water Fish Reveals Divergent Intestinal Characteristics and Microbiome Assembly

**DOI:** 10.3390/ani16060895

**Published:** 2026-03-12

**Authors:** Xiao Yang, Kaixuan Liu, Wei Yang, Tianzhi Jin, Jiahong Li, Zhijian Wang, Fang Li

**Affiliations:** 1 Integrative Science Center of Germplasm Creation in Western China (Chongqing) Science City, MOE Key Laboratory of Freshwater Fish Reproduction and Development, School of Life Sciences, Southwest University, Chongqing 400715, China; 2 Integrative Science Center of Germplasm Creation in Western China (Chongqing) Science City, MOE Key Laboratory of Freshwater Fish Reproduction and Development, College of Fisheries, Southwest University, Chongqing 400715, China; 3 School of Agronomy, Xinjiang Hetian College, Hetian 848000,China

**Keywords:** *Schizothorax*, reciprocal hybridization, intestinal microbiota, digestive enzymes, phenotypic integration, intestinal plasticity

## Abstract

Hybridization is pivotal for germplasm innovation, yet how reciprocal crossing regulates digestive characteristics in sub-cold-water fish remains unclear. This study systematically compared differences in intestinal morphology, physiological function, and microbial community assembly among herbivorous *Schizothorax prenanti*, carnivorous *S. davidi*, and their reciprocal hybrids using histological analysis, digestive enzyme assays, and 16S rRNA sequencing. Results indicated that parental intestinal characteristics were highly consistent with their feeding habits. Orthogonal hybrids exhibited a mosaic phenotype, combining the maternal muscular gut structure with high paternal-like lipase activity, and were characterized by an enrichment of the potential probiotic *Lactococcus*. In contrast, reciprocal hybrids presented a mismatch between morphology and function: despite developed hindgut folds, key digestive enzyme activities were low, and the gut microbiota was dominated by environmental bacteria such as *Methylobacterium*. Our findings indicate a spatially dependent assembly dynamic: the host genetic background strongly drives microbiome divergence in the anterior segments (foregut and midgut), whereas the long-term administration of a standardized diet ultimately promotes structural convergence in the hindgut. The orthogonal cross yielded a phenotype characterized by an apparent co-occurrence of specific host enzymes and distinct microbiota, suggesting an inferred physiological potential for lipid digestion that requires further multi-omics validation. These findings provide preliminary insights into the associations between genetic background and intestinal traits, providing a theoretical basis for the targeted breeding of *Schizothorax* species.

## Simple Summary

Hybridization is a fundamental approach in aquaculture breeding, yet the specific effects of reciprocal crossing on the intestinal phenotype of sub-cold-water fish remain under-explored. This study characterized the intestinal morphology, digestive enzyme profiles, and microbiome composition in the hybrid offspring of a carnivorous species (*Schizothrax davidi*) and an herbivorous species (*Schizothorax prenanti*). We observed that the direction of hybridization resulted in distinct trait combinations. The orthogonal hybrids (paternal carnivore × maternal herbivore) exhibited a mosaic phenotype, combining the muscular gut structure of the mother with the high lipase activity of the father, accompanied by an enrichment of *Lactococcus*. In contrast, the reciprocal hybrids displayed a different structural–physiological pattern, characterized by extensive fold development but lower amylase activity, and a microbiome composition with a higher proportion of environmental taxa. These findings provide a detailed description of how hybridization differentially remodels the digestive tract and microbial assembly in sub-cold-water fish, highlighting the importance of crossing direction in shaping biological traits.

## 1. Introduction

The sustainable development of modern aquaculture relies heavily on the continuous innovation of germplasm resources. As wild capture fisheries can no longer meet the growing global demand for aquatic products, hybridization technology has been widely applied since the 1980s as a key breeding strategy to develop new varieties with superior traits, addressing the continuous global increase in demand for high-quality animal protein [1,2]. The core biological basis of this application lies in heterosis, a complex phenomenon in which hybrid offspring exhibit performance superior to that of their parents in various aspects, such as growth rate, feed efficiency, and stress resistance [3,4,5]. Among these, the improvement of growth performance is one of the most scrutinized phenotypes of heterosis, a process that fundamentally depends on the efficient digestion and absorption of food. Therefore, investigating the adaptive remodeling of the digestive system in hybrid offspring is a critical physiological link for understanding the mechanisms underlying heterosis formation [6,7].

The functional phenotype of the digestive system is profoundly shaped by the host’s genetic background. To adapt to their specific nutritional niches, fish with different feeding habits have evolved distinct digestive strategies, which are manifested at multiple levels, including intestinal morphological structure, digestive enzyme activity profiles, and the symbiotic gut microbial community [8]. In particular, the gut microbiome, regarded as the host’s “second genome,” plays an indispensable role in the host’s nutrient metabolism, immune homeostasis, and environmental adaptation. Existing studies indicate that hybridization events can significantly drive the remodeling of the offspring’s gut microbiota. For instance, relevant studies on cyprinid fishes have revealed that hybrid offspring may construct microbial communities that are more diverse or structurally distinct from those of their parents; furthermore, this remodeling often exhibits a significant tendency towards a specific parent, suggesting that the host genome screens and dominates the assembly of the core microbiota through specific mechanisms [9,10]. However, a critical theoretical bottleneck limits the in-depth interpretation of these findings. In the distant hybridization of fish, asymmetry in reciprocal cross results is prevalent; specifically, reciprocal cross combinations often fail to yield viable offspring due to issues such as nucleo-cytoplasmic conflict or genomic incompatibility [11]. Consequently, the vast majority of existing studies have been restricted to hybridization models based on a single direction (usually the orthogonal cross), failing to establish comparable reciprocal populations. This makes it difficult to systematically distinguish the independent contributions of maternal effects and nuclear genetic effects in shaping the intestinal microecology and digestive phenotypes.

The genus *Schizothorax* represents an endemic and economically important taxon in the Qinghai–Tibet Plateau and its adjacent waters, which has evolved significant dietary divergence during long-term adaptive evolution [12]. For instance, *S. prenanti* possesses a horny sheath on its lower jaw and primarily feeds by scraping periphytic algae, making it a typical herbivorous species. In contrast, *S. davidi* features a blunt snout and mainly preys on benthic invertebrates, characterizing it as a carnivorous-leaning species [13]. These two species, exhibiting distinct feeding habits yet sharing sympatric distribution, provide an ideal model for investigating dietary inheritance and digestive adaptation. Crucially, unlike widely studied warm-water fishes such as tilapia and common carp, *S.* species are plateau sub-cold-water fishes that inhabit low-temperature and oligotrophic environments. They are characterized by low basal metabolic rates, low baseline enzyme activities, and a fragile balance of energy budget [14]. When herbivorous and carnivorous genomes are combined under such demanding physiological backgrounds, the remodeling efficiency of the digestive system in their hybrid offspring directly determines whether they can acquire sufficient energy to support survival and growth under low-metabolic constraints. This endows the present study with unique ecophysiological significance.

To address the aforementioned research gaps, this study constructed reciprocal cross combinations of *S*. *prenanti* and *S*. *davidi*, two species with significantly divergent feeding habits. Given the extreme dietary divergence between the herbivorous and carnivorous parents, we hypothesized that the reciprocal hybrids would not exhibit a simple intermediate digestive phenotype, but rather an asymmetric inheritance in intestinal morphology, enzyme activity, and microbiota assembly depending on the hybridization direction. To characterize these intestinal traits and establish a foundational baseline, this study aimed to: (1) compare the differences in intestinal histomorphology, digestive enzyme activities, and microbial community structures among the parents and their hybrids; (2) evaluate the phenotypic similarities between the reciprocal hybrids and their parental species; and (3) explore the potential associations between core functional microbiota and specific nutritional substrates to resolve these issues, this study integrated histology, enzymology, and 16S rRNA gene sequencing technologies. Through precise segmental sampling and analysis of the foregut, midgut, and hindgut of both parents and reciprocal hybrids, combined with biostatistical methods, the multidimensional interplay among host genetics, intestinal morphology, digestive physiology, and microecology was comprehensively analyzed.

This study holds multiple scientific values and broad application prospects. Theoretically, it systematically analyzes the transgenerational inheritance patterns and microecological mechanisms of digestive traits for the first time in plateau sub-cold-water fish using a viable reciprocal cross model, providing foundational data on the intestinal characteristics and microbial composition of these species, which contributes to our understanding of digestive profiles in hybrid fish. Practically, the results will help evaluate the underlying digestive capacity and physiological potential of hybrid offspring to artificial compound feeds. This provides a precise physiological basis and data support for the exploitation and utilization of *Schizothorax* germplasm resources, the selective breeding of targeted hybrid varieties, the development of specialized feed formulations, and the formulation of healthy farming strategies based on microecological regulation, thereby promoting the sustainable development of plateau-characteristic fisheries.

## 2. Materials and Methods

### 2.1. Ethics Statement

All animal procedures in this research were conducted in accordance with the Guidelines for the Care and Use of Laboratory Animals in China and approved by the Southwest University Animal Ethics Committee (permit No. IACUC-20230928-01). MS-222 anesthetic was used during sampling to minimize the suffering of the experimental fish.

### 2.2. Experimental Fish and Rearing Management

**Figure 1:**
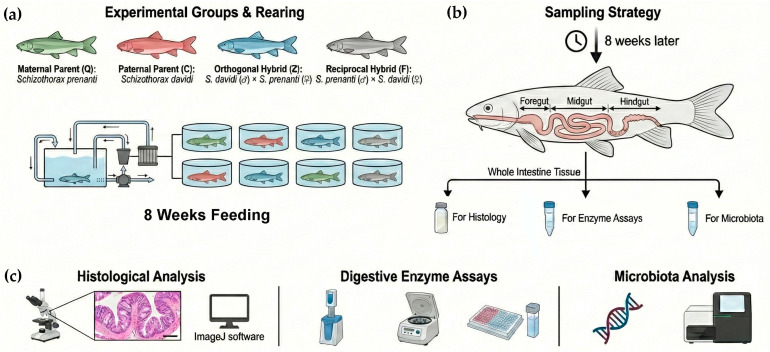
Schematic diagram of the experimental design and analytical procedures. (**a**) Experimental setup involving four groups: *S. prenanti* (Q), *S. davidi* (C), orthogonal hybrid (Z), and reciprocal hybrid (F). Fish were reared in a recirculating system for 8 weeks. (**b**) Sample collection workflow. Intestinal tissues were sampled for histology, enzyme assays, and microbiota analysis. (**c**) Downstream analyses including histological observation (H&E staining), digestive enzyme activity determination, and gut microbiota profiling.

The experimental fish included *S*. *prenanti* (Q), *S*. *davidi* (C), their orthogonal hybrid (*S. davidi* ♂ × *S. prenanti* ♀, Z), and their reciprocal hybrid (*S. prenanti* ♂ × *S. davidi* ♀, F). All fish were obtained from the Integrative Science Center of Germplasm Creation in Western China, Southwest University, Chongqing, China. To ensure environmental consistency, and eliminate confounding effects from divergent early microbial colonization, fertilized eggs were transported to the laboratory. The rearing system was disinfected with potassium permanganate (KMnO_4_) prior to use, and aerated tap water was used for aquaculture to ensure consistent initial environmental conditions. These fish were reared to adulthood (2 years) in this controlled environment. Crucially, following an initial exogenous feeding phase with egg yolk and *Artemia* nauplii, all groups were exclusively fed the exact same commercial diet formulation (adjusted only for pellet size to accommodate growth) that was subsequently utilized in the feeding trial. Prior to the experiment, all fish were acclimated in a recirculating aquaculture system for 2 weeks. Fish of uniform size (mean initial body weight: 58 ± 1.42 g) were selected after strict visual and behavioral inspections to ensure they were active, feeding normally, and free of visible lesions or deformities. The selected fish were randomly distributed into 12 rearing tanks (109 cm × 67.5 cm × 35 cm, water depth: 30 cm) across three independent recirculating aquaculture systems (RAS), with 15 fish per tank, resulting in a stocking density of approximately 0.068 fish/L. Each independent RAS contained four tanks, with one replicate tank assigned to each of the four treatment groups. During the experimental period, fish were fed a commercial diet (crude protein ≥ 40%, crude lipid ≥ 4.2%) twice daily at 09:00 and 17:00. Water temperature was maintained at 22 ± 1 °C, dissolved oxygen concentration was kept above 6.0 mg/L, ammonia nitrogen concentration was below 0.05 mg/L, pH was maintained at 7.5 ± 0.2, and the photoperiod was set at 12L:12D (Figure 1a).

### 2.3. Sample Collection

After the 8-week feeding trial, all experimental fish were fasted for 24 h. Two fish of uniform size were randomly netted from each of the three replicate tanks per treatment group (totaling six fish per group). These fish were deeply anesthetized with 200 mg/L MS-222 (Tricaine methanesulfonate, Sigma-Aldrich, St. Louis, MO, USA). Throughout the entire sampling process, strictly sterilized instruments and RNase/DNase-free consumables were used to prevent any environmental microbial contamination. Fish were dissected under sterile conditions to isolate the complete intestine. The anatomical sampling sites for all tissues were kept strictly consistent across all different genetic groups and individual fish. For histological analysis, tissue segments from identical anatomical locations of the foregut, midgut, and hindgut were excised and immediately fixed in 4% paraformaldehyde (*n* = 5). For enzyme activity analysis, an equal-proportion mixture of tissue segments from the foregut, midgut, and hindgut was collected, rinsed with 0.9% saline, placed in sterile cryotubes, and immediately snap-frozen in liquid nitrogen (*n* = 5). For microbiological analysis, contents and mucosal tissues from the foregut, midgut, and hindgut were separately collected from all six sampled individuals (*n* = 6 per group), placed in sterile cryotubes, and immediately snap-frozen in liquid nitrogen. All samples were subsequently stored at −80 °C until analysis (Figure 1b).

### 2.4. Histological Examination

Fixed intestinal tissues were embedded in paraffin. Continuous cross-sections (6 μm) were prepared (Leica RM2235, Leica Camera, Wetzlar, Germany) and stained with hematoxylin and eosin (H&E). Stained sections were photographed using an optical microscope (Nikon Eclipse Ci-L, Nikon Corporation, Melville, NY, USA). Morphometric parameters (fold height (FH), fold width (FW), inner circular muscle thickness (ICM), and outer longitudinal muscle thickness (OLM)) were measured using ImageJ version 1.54f (Detailed measurement schematic is shown in Supplementary Figure S1). For each fish (*n* = 5 per group), approximately 10 intact folds and muscle layers across two random fields of view were measured and averaged to yield a single biological replicate value (Figure 1c).

### 2.5. Digestive Enzyme Activity Assay

Intestinal tissue samples were accurately weighed and homogenized in ice-cold physiological saline at a ratio of 1:9 (*w*/*v*) under ice-bath conditions. The homogenate was centrifuged at 2500 rpm for 10 min at 4 °C, and the supernatant was collected for enzyme activity assays. The activities of α-amylase, lipase, and trypsin were determined using commercial kits (Nanjing Jiancheng Bioengineering Institute, Nanjing, China). Total protein concentration was measured using the Coomassie Brilliant Blue assay kit (Nanjing Jiancheng Bioengineering Institute, Nanjing, China). All procedures were strictly performed according to the manufacturers’ instructions. The definition of enzyme activity units (U) referred to the specific instructions of each kit (Figure 1c).

### 2.6. DNA Extraction and PCR Amplification

Total genomic DNA was extracted from intestinal content samples using the E.Z.N.A.^®^ Soil DNA Kit (Omega Bio-tek, Norcross, GA, USA) according to the manufacturer’s instructions. DNA concentration and purity were determined using a NanoDrop 2000 spectrophotometer (Thermo Scientific, Wilmington, DE, USA), and DNA integrity was checked by 1% agarose gel electrophoresis. The V3–V4 hypervariable region of the bacterial 16S rRNA gene was amplified using specific primers with barcodes: 338F (5′-ACTCCTACGGGAGGCAGCAG-3′) and 806R (5′-GGACTACHVGGGTWTCTAAT-3′). The PCR reaction mixture (20 μL) contained 4 μL of 5× TransStart FastPfu Buffer, 2 μL of 2.5 mM dNTPs, 0.8 μL of forward primer (5 μM), 0.8 μL of reverse primer (5 μM), 0.4 μL of TransStart FastPfu DNA Polymerase (TransGen Biotech, Beijing, China), 10 ng of template DNA, and ddH_2_O to volume. The amplification program was as follows: initial denaturation at 95 °C for 3 min; followed by 27 cycles of denaturation at 95 °C for 30 s, annealing at 55 °C for 30 s, and extension at 72 °C for 30 s; and a final extension at 72 °C for 10 min (T100 Thermal Cycler, Bio-Rad, Hercules, CA, USA). PCR products were checked by 2% agarose gel electrophoresis, purified using the PCR Clean-Up Kit (Axygen, Union City, CA, USA), and quantified using a Qubit 4.0 Fluorometer (Thermo Fisher Scientific, Waltham, MA, USA). Furthermore, to definitively rule out the possibility of reagent or environmental contamination during the laboratory procedures, DNA extraction blanks and PCR negative controls (using nuclease-free water as templates) were processed concurrently with all samples. Following PCR amplification, no visible bands were detected in these negative controls via agarose gel electrophoresis, and subsequent library construction for these controls failed due to insufficient DNA yield, thereby confirming the absence of ‘kitome’ or systematic laboratory contamination.

### 2.7. Bioinformatics Analysis

Purified amplicons were subjected to paired-end sequencing on an Illumina Nextseq 2000 platform (Illumina, San Diego, CA, USA). Raw sequencing data were first quality-controlled using fastp software (version 0.19.6) to filter low-quality reads and then merged using FLASH software (version 1.2.11). Based on the QIIME2 pipeline (version 2022.2), the DADA2 plugin was used to denoise the optimized sequences, remove chimeras and singletons, and generate amplicon sequence variants (ASVs). ASV sequences were aligned against the SILVA 16S rRNA database (version 138.2), and taxonomic annotation was performed using the Naive Bayes classifier. Sequences annotated as chloroplasts or mitochondria were removed. To eliminate the effect of differences in sequencing depth, the sequence number of all samples was rarefied to the minimum sample sequence number (e.g., 20,000 reads).

### 2.8. Statistical Analysis

Alpha diversity indices (Chao1, Shannon, Simpson) of the microbial community were calculated using mothur software (version 1.30.2). Principal Coordinate Analysis (PCoA) based on Bray–Curtis distances and Permutational Multivariate Analysis of Variance (PERMANOVA) were performed within the QIIME2 environment to evaluate Beta diversity differences. Linear Discriminant Analysis Effect Size (LEfSe, http://huttenhower.sph.harvard.edu/lefse/, accessed on 10 March 2026) was used to identify biomarkers with significant differences between groups (LDA threshold > 2.0, *p* < 0.05). Based on the top 30 abundant genera in each group, Spearman correlation coefficients were calculated, and relationships with |*r*| > 0.6 and *p* < 0.05 were selected to construct co-occurrence networks. Network visualization and topological property calculations were performed using Gephi software (version 0.9.2). Spearman correlation heatmaps between core bacterial genera and digestive enzyme activities were plotted using the pheatmap package in R language (version 4.1.3). All physiological and biochemical data (enzyme activities, histological parameters) are presented as “Mean ± Standard Deviation” (Mean ± SD). Statistical analyses were performed using SPSS software (version 26.0, IBM Corp., Armonk, NY, USA). Data were first tested for normality using the Shapiro–Wilk test and for homogeneity of variance using Levene’s test. If conditions were met, one-way Analysis of Variance (ANOVA) followed by Tukey’s HSD post hoc test was used to examine differences between groups. Data that did not satisfy normal distribution were analyzed using the Kruskal–Wallis non-parametric test. The significance level was set at *p* < 0.05. Additionally, the Benjamini–Hochberg FDR correction was applied to all Spearman correlation *p*-values to control for false positives.

## 3. Results

### 3.1. Intestinal Histomorphological Characteristics

**Figure 2:**
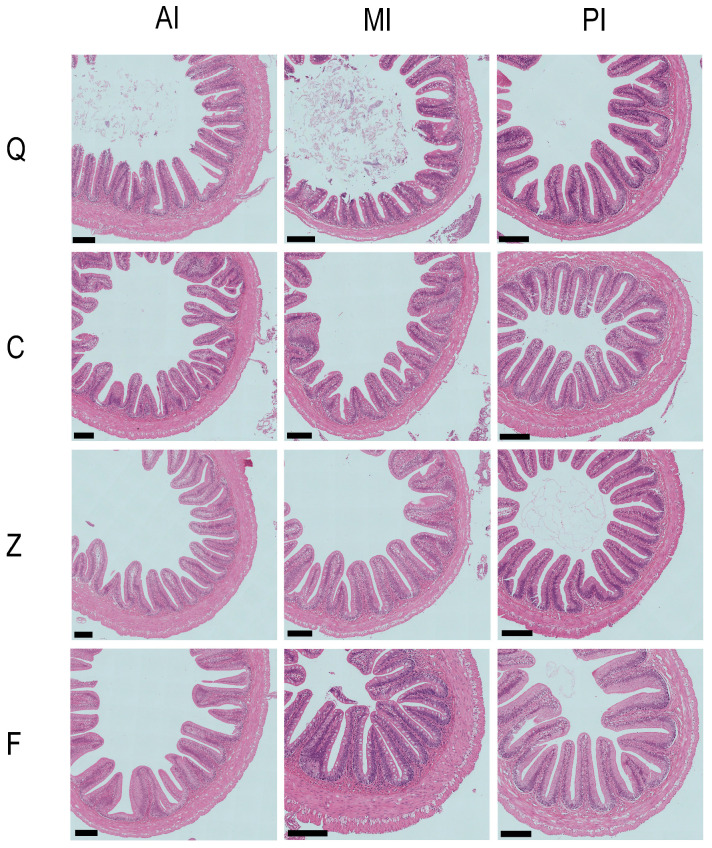
Histological microstructures of different intestinal segments in parental and hybrid *Schizothorax* groups. Representative photomicrographs of hematoxylin and eosin (H&E)-stained transverse sections. The panel is organized as follows: Rows 1 to 4 represent *S. prenanti* (Q), *S. davidi* (C), the orthogonal hybrid (Z), and the reciprocal hybrid (F), respectively; Columns 1 to 3 represent the anterior intestine (AI), middle intestine (MI), and posterior intestine (PI), respectively. Black scale bar = 200 μm.

Measurements of intestinal tissue sections revealed significant differences in intestinal morphological structures among *S. prenanti* (Q), *S. davidi* (C), and their hybrid offspring (Table 1, Figure 2).

In the foregut, *S. davidi* (C) exhibited the most developed mucosal folds, with both fold height (FH) and fold width (FW) being significantly higher than those of *S. prenanti* (Q) and the two hybrid groups (*p* < 0.05). Regarding the muscle layers, the inner circular muscle (ICM) of *S. prenanti* (Q) was the most developed, being significantly thicker than that of the other three groups (*p* < 0.05). For the orthogonal hybrids (Z), the fold morphological parameters were similar to those of *S. prenanti* (Q) and reciprocal hybrids (F), and all were significantly lower than those of *S. davidi*; however, in terms of outer longitudinal muscle (OLM) thickness, the Z group displayed higher values similar to *S. prenanti* (Q), which were significantly thicker than those of *S. davidi* (C, *p* < 0.05).

Morphological differences in the midgut followed a different pattern. *S. davidi* (C) maintained the highest FH and FW (*p* < 0.05). The FH of orthogonal hybrids (Z) was the lowest, being significantly lower than that of *S. davidi* (C). Regarding muscle layer thickness, the ICM showed distinct transitional characteristics: it was thickest in *S. prenanti* (Q) and thinnest in *S. davidi* (C), while the thickness in hybrids (Z and F) was intermediate between the two parental species and significantly higher than that in *S. davidi* (*p* < 0.05). For OLM thickness, both Z and F groups exhibited higher values that were not significantly different from *S. prenanti* (Q) but were significantly thicker than those of *S. davidi* (C, *p* < 0.05).

In the hindgut, morphological differences among groups were relatively minor. No significant differences were detected in FW or OLM thickness across all groups (*p* > 0.05). However, regarding FH, the reciprocal hybrids (F) exhibited the highest value, which was significantly higher than that of *S. prenanti* (Q) and orthogonal hybrids (Z, *p* < 0.05). Similarly, the ICM thickness of reciprocal hybrids (F) was also at a higher level, being significantly thicker than that of *S. davidi* (C), while the Q and Z groups showed intermediate levels with no significant difference.

Overall, the intestinal characteristics of *S. davidi* were primarily characterized by developed mucosal fold structures, whereas *S. prenanti* possessed more developed muscle layers. The orthogonal hybrids (Z) more closely resembled *S. prenanti* in fold morphology but retained the developed muscle layer characteristics of *S. prenanti*, with certain parameters (such as midgut ICM) being intermediate between *S. prenanti* and *S. davidi*.

### 3.2. Analysis of Intestinal Digestive Enzyme Activities

**Figure 3:**
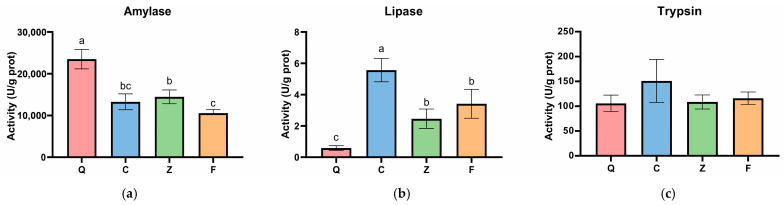
Comparison of intestinal digestive enzyme activities among parental and hybrid *Schizothorax* groups. (**a**) Amylase activity; (**b**) Lipase activity; (**c**) Trypsin activity. Data are presented as mean ± SD. Different lowercase letters above the bars indicate significant differences among groups (*p* < 0.05). Abbreviations: Q: *S. prenanti*; C: *Schizothorax davidi*; Z: Orthogonal hybrid (*S. davidi* ♂ × *S. prenanti* ♀); F: Reciprocal hybrid (*S. prenanti* ♂ × *S. davidi* ♀).

To evaluate the potential impact of alterations in the intestinal microbiota on host digestive function, the activities of three key digestive enzymes were determined (Figure 3). The results indicated that, with the exception of trypsin, the activities of amylase and lipase exhibited significant differences among *Schizothorax* groups with different genetic backgrounds. Specifically, amylase activity was highest in *S. prenanti* (group Q), reaching 23,500 ± 2320 U/g prot, which was significantly higher than that in the other three groups (*p* < 0.05). The amylase activity of the orthogonal hybrids (group Z) was 14,460 ± 1640 U/g prot, significantly higher than that of the reciprocal hybrids (10,540 ± 870 U/g prot). The paternal *S. davidi* showed an intermediate level between the two hybrid groups, with no significant differences observed compared to either hybrid group (*p* > 0.05). Regarding lipase activity, *S. davidi* (group C) exhibited the highest level, reaching 5.56 ± 0.75 U/g prot, which was significantly higher than that of all other groups (*p* < 0.05). Notably, the lipase activities of the hybrid offspring (groups Z and F) were 2.46 ± 0.62 U/g prot and 3.41 ± 0.92 U/g prot, respectively. No statistical difference was found between the two hybrid groups, but both were significantly higher than that of *S. prenanti* (group Q, 0.58 ± 0.15 U/g prot). Furthermore, no significant differences were detected in trypsin activity among the four experimental groups (*p* > 0.05).

### 3.3. Alpha Diversity Analysis of the Intestinal Microbiota

#### 3.3.1. Spatial Distribution Characteristics of the Intestinal Microbiota

**Figure 4:**
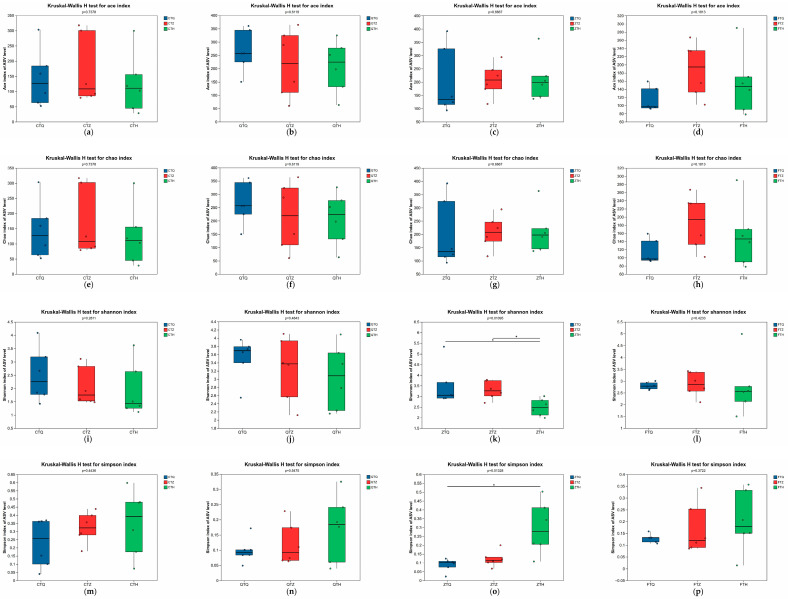
Spatial variation of Alpha diversity indices along the intestinal tract in parental and hybrid *Schizothorax*. The panels illustrate the comparisons of Alpha diversity indices among the foregut, midgut, and hindgut within each group. (**a**–**d**) The ACE index for *S. davidi* (C), *S. prenanti* (Q), Orthogonal hybrid (Z), and Reciprocal hybrid (F), respectively. (**e**–**h**) The Chao1 index for C, Q, Z, and F groups. (**i**–**l**) The Shannon index for C, Q, Z, and F groups. (**m**–**p**) The Simpson index for C, Q, Z, and F groups. Asterisks (*) indicate significant differences between intestinal segments within the same group (*p* < 0.05).

The Alpha diversity of the microbiota in the foregut, midgut, and hindgut of the same *Schizothorax* group was compared using the Kruskal–Wallis H test (Figure 4). The results showed that the spatial distribution of the intestinal microbiota in *S. prenanti* (Q), *S. davidi* (C), and the reciprocal hybrids (F) was relatively uniform; no significant differences were observed in their Chao1 richness indices or Shannon diversity indices among the three intestinal segments (*p* > 0.05). In contrast, the orthogonal hybrids (Z) exhibited significant spatial heterogeneity. The Shannon index of the Z group differed significantly among intestinal segments (Figure 4k), characterized by significantly higher diversity in the foregut and midgut compared to the hindgut (*p* < 0.05).

#### 3.3.2. Comparison of Intestinal Microbiota Differences Among Different Schizothorax Groups

**Figure 5:**
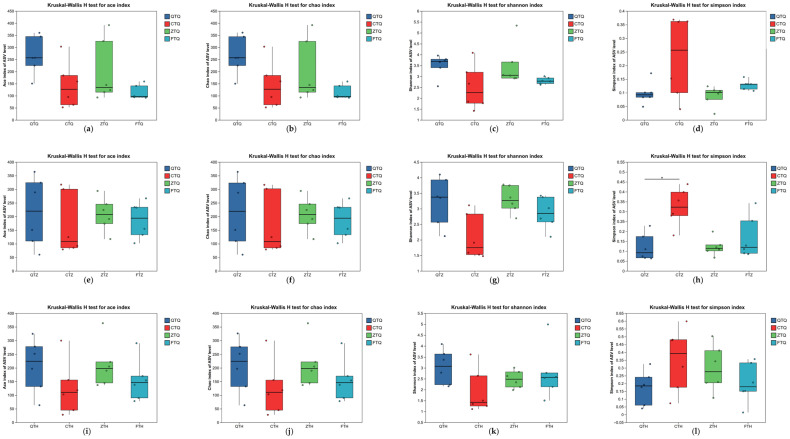
Comparison of Alpha diversity indices of intestinal microbiota among parental and hybrid *Schizothorax* groups across different intestinal segments. The box plots display the ACE, Chao1, Shannon, and Simpson indices in the (**a**–**d**) Foregut, (**e**–**h**) Midgut, and (**i**–**l**) Hindgut, respectively. The horizontal line within the box represents the median, and the whiskers indicate the minimum and maximum values. Asterisks (*) indicate significant differences between groups (*p* < 0.05). Abbreviations: Q: *S. prenanti*; C: *S. davidi*; Z: Orthogonal hybrid (*S. davidi* ♂ × *S. prenanti* ♀); F: Reciprocal hybrid (*S. prenanti* ♂ × *S. davidi* ♀).

Alpha diversity differences among the four experimental groups (Q, C, Z, F) were further compared while controlling for the intestinal segment variable (Figure 5). In the foregut, significant differences were observed among groups; *S. prenanti* (Q) and the orthogonal hybrids (Z) maintained relatively high community diversity. In the midgut, Alpha diversity among groups did not reach a level of statistical significance (*p* > 0.05); however, in terms of numerical trends, the orthogonal hybrids (Z) and *S. prenanti* (Q) remained higher than *S. davidi* (C). In the hindgut, significant differences were again exhibited among groups (*p* < 0.05), with *S. prenanti* (Q) and the orthogonal hybrids (Z) continuing to maintain a relatively complex microbial community structure.

### 3.4. Beta Diversity Analysis of the Intestinal Microbiota

**Figure 6:**
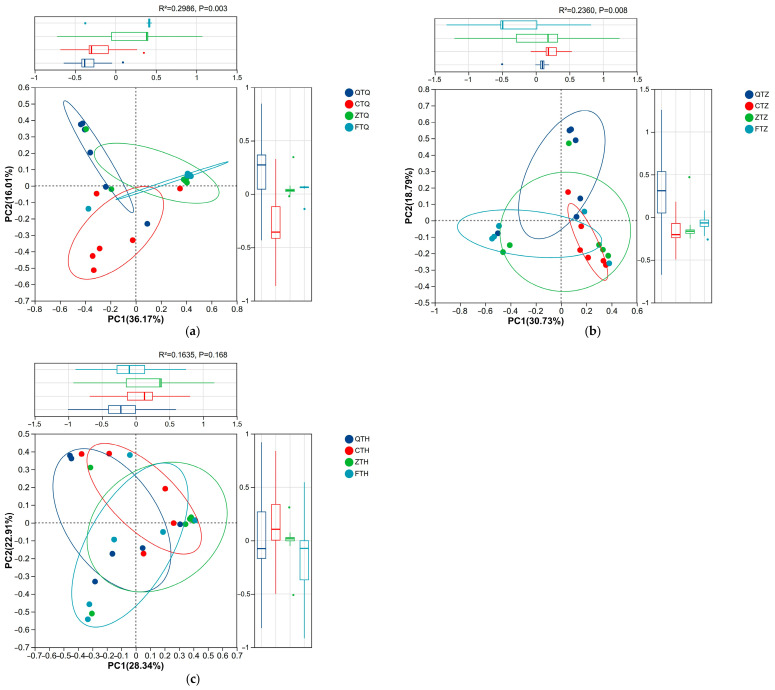
Beta diversity analysis of intestinal microbiota communities among parental and hybrid *Schizothorax* groups across different intestinal segments. Principal Coordinate Analysis (PCoA) plots based on Bray–Curtis dissimilarity matrices illustrating the clustering of microbiota samples in the (**a**) Foregut, (**b**) Midgut, and (**c**) Hindgut. Each point represents an individual sample, and colors indicate different fish groups. Ellipses represent 95% confidence intervals. The percentage on each axis indicates the proportion of total variance explained by that component. Statistical significance of group separation was determined using PERMANOVA (Adonis), Statistical validation indices for group separation are provided in Supplementary Table S1. Abbreviations: Q: *S. prenanti*; C: *S. davidi*; Z: Orthogonal hybrid (*S. davidi* ♂ × *S. prenanti* ♀); F: Reciprocal hybrid (*S. prenanti* ♂ × *S. davidi* ♀).

Principal Coordinate Analysis (PCoA) based on Bray–Curtis distance matrices and Permutational Multivariate Analysis of Variance (PERMANOVA) results showed that the microbial community structure in the foregut differed significantly between parents and hybrid offspring (Figure 6). Host genetic background explained 29.9% of the total variation in the foregut microbiota, and the microbial community structure was not randomly distributed among groups. Further analysis of the distribution differences among groups along the PCoA axes revealed that PC1 highlighted a differentiation trend between hybrid offspring and parents. Orthogonal hybrids (Z) and reciprocal hybrids (F) were distributed mainly on the positive semi-axis of PC1, while the parental *S. prenanti* (Q) and *S. davidi* (C) were distributed mainly on the negative semi-axis. The difference between orthogonal hybrids (Z) and the maternal parent (Q) on the PC1 axis showed a significant statistical trend (*p* < 0.05), indicating that the microbial community structure of hybrid offspring had deviated from the maternal parent in terms of primary community characteristics. PC2 significantly distinguished the paternal parent from the hybrid offspring. On the PC2 axis, orthogonal hybrids (Z) showed significant statistical differences from the paternal *S. davidi* (C) (*p* < 0.05). Similarly, reciprocal hybrids (F) also differed significantly from the paternal parent (C) on PC2 (*p* < 0.05). Beta diversity analysis of the foregut microbiota indicated that the microbial community structure of the hybrid offspring (Z and F) had undergone significant remodeling; they differed from the paternal parent and also exhibited a trend of deviation from the maternal parent, forming a unique microbial community structure.

The microbial community structure in the midgut also differed significantly between parents and hybrid offspring. Host genetic background explained 23.6% of the total variation in the midgut microbiota. Further analysis of the distribution differences along the PCoA axes revealed a genetic bias pattern distinct from that of the foregut. The distribution of experimental groups on the PC1 axis overlapped relatively well, and no significant statistical differences were detected among groups (*p* > 0.05). PC2 revealed a significant paternal bias in the midgut microbiota. On the PC2 axis, orthogonal hybrids (Z) showed significant statistical differences from the maternal *S. prenanti* (Q) (*p* < 0.05). However, the difference between orthogonal hybrids (Z) and the paternal *S. davidi* (C) on PC2 was not significant (*p* > 0.05), and the two groups were closer in distribution patterns. Furthermore, *S. prenanti* and *S. davidi* also differed significantly on PC2 (*p* < 0.05), further confirming that the PC2 axis primarily reflected genetic differentiation between parents. Beta diversity analysis of the midgut microbiota indicated that the microbial community structure of orthogonal hybrids (Z) exhibited a significant characteristic of paternal bias (towards C) on the principal PC2 axis, while significantly deviating from the maternal parent (Q).

Unlike the significant differentiation observed in the foregut and midgut, no significant differences were detected in the hindgut microbial community structure between parents and hybrid offspring (Adonis R^2^ = 0.163, *p* = 0.168), indicating a weak influence of host genetic background on the hindgut microbiota. PCoA analysis further confirmed that the distribution of all groups overlapped highly on major dimensions with no statistical differences (*p* > 0.05), reflecting the convergence of the hindgut microbiota across groups.

### 3.5. Taxonomic Composition Analysis of Intestinal Microbiota

#### 3.5.1. Taxonomic Composition at the Phylum Level

**Figure 7:**
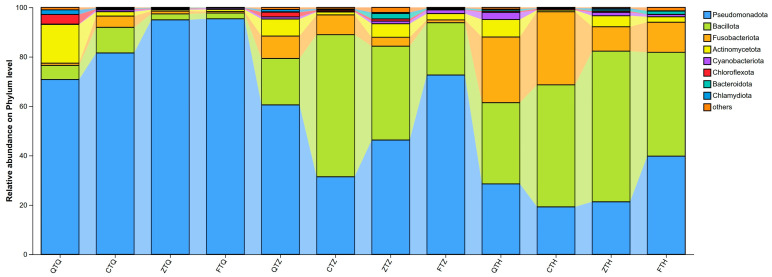
Taxonomic composition of intestinal microbiota at the phylum level across different intestinal segments in parental and hybrid *Schizothorax* groups. The stacked bar plot displays the mean relative abundance of the top 10 dominant bacterial phyla in the foregut, midgut, and hindgut. Each bar represents the average of the biological replicates for each group. Taxa with a relative abundance of less than 1% are grouped as “Others”. Abbreviations: Q: *S. prenanti*; C: *S. davidi*; Z: Orthogonal hybrid (*S. davidi* ♂ × *S. prenanti* ♀); F: Reciprocal hybrid (*S. prenanti* ♂ × *S. davidi* ♀).

The microbial communities in different intestinal segments of parental and hybrid *Schizothorax* were annotated and analyzed at the phylum level (Figure 7). The results showed that samples from all groups were dominated by three core phyla: Pseudomonadota (formerly Proteobacteria), Bacillota (formerly Firmicutes), and Fusobacteriota (formerly Fusobacteria). These three taxa accounted for the vast majority of total sequencing reads, constituting the main framework of the *Schizothorax* intestinal microbiota. Additionally, Cyanobacteriota, Actinomycetota, and Bacteroidota were present as minor taxa with low abundance across different groups.

In the foregut, the microbial community exhibited a single-dominance pattern characterized by an absolute prevalence of Pseudomonadota. Specifically, the abundance of Pseudomonadota in hybrid offspring groups was extremely high, with relative abundances in reciprocal hybrids (FTQ) and orthogonal hybrids (ZTQ) reaching 95.37% and 94.97%, respectively. In contrast, the abundance of Pseudomonadota in parental groups was slightly lower: 81.55% in *S. davidi* (CTQ) and 70.80% in *S. prenanti* (QTQ). Notably, Bacillota accounted for a certain proportion (10.39%) in the paternal *S. davidi* (CTQ), whereas it was lower in other groups (2.3–5.7%). Furthermore, the maternal *S. prenanti* (QTQ) exhibited specific colonization of Fusobacteriota in the foregut, with a relative abundance of 12.51%, significantly higher than that in the other three groups.

In the midgut, the microbial community structure underwent drastic inter-group differentiation and succession. The dominance of Pseudomonadota weakened in most groups, while the proportions of Bacillota and Fusobacteriota increased. The paternal *S. davidi* (CTZ) exhibited a characteristic dominance of Bacillota, with its abundance rising to 57.54%. In sharp contrast, Fusobacteriota became the most dominant phylum in the midgut microbiota of orthogonal hybrids (ZTZ), with a relative abundance as high as 47.04%, while their Pseudomonadota proportion dropped to the lowest among all groups (46.30%). Reciprocal hybrids (FTZ) maintained a relatively high abundance of Pseudomonadota (72.62%), accompanied by 21.11% Bacillota, displaying transitional characteristics intermediate between the parents.

In the hindgut, Bacillota became the major dominant phylum. Descriptively, orthogonal hybrids (ZTH) maintained the highest abundance of Bacillota in the hindgut, reaching 60.98%, which was higher than their levels in the foregut and midgut. The Bacillota abundance in the paternal parent (CTH) and reciprocal hybrids (FTH) also reached 49.40% and 42.05%, respectively. Meanwhile, Fusobacteriota maintained relatively high proportions in the parental groups (QTH: 28.43%, CTH: 25.00%), whereas its proportions were relatively lower in the hybrid offspring (ZTH: 11.85%, FTH: 11.83%). Overall, the proportion of Pseudomonadota in the hindgut decreased substantially to the range of 19–40%.

#### 3.5.2. Taxonomic Composition at the Genus Level

**Figure 8:**
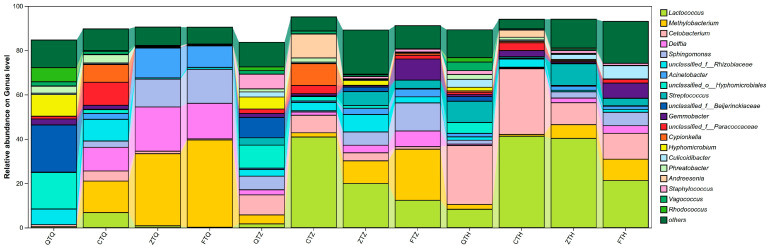
Taxonomic composition of intestinal microbiota at the genus level across different intestinal segments in parental and hybrid *Schizothorax* groups. The stacked bar plot displays the mean relative abundance of the top 20 dominant bacterial genera in the foregut, midgut, and hindgut. Each bar represents the average of the biological replicates for each group. Taxa with a relative abundance of less than 1% are grouped as “Others”. Abbreviations: Q: *S. prenanti*; C: *S. davidi*; Z: Orthogonal hybrid (*S. davidi* ♂ × *S. prenanti* ♀); F: Reciprocal hybrid (*S. prenanti* ♂ × *S. davidi* ♀).

Taxonomic classification was refined to the genus level to analyze the top 20 dominant bacterial genera by relative abundance (Figure 8). The results showed that the *Schizothorax* intestinal microbial community was primarily dominated by core genera such as *Methylobacterium*, *Lactococcus*, *Cetobacterium*, *Delftia*, and *Sphingomonas*. These dominant genera exhibited significant distributional differences across genetic backgrounds and intestinal segments, constituting the unique microbial community characteristics of each experimental group.

In the foregut, hybrid offspring (Z and F) exhibited dominant microbiota patterns similar to the paternal parent (C), primarily dominated by *Methylobacterium*, *Delftia*, and *Sphingomonas*. Specifically, the relative abundance of *Methylobacterium* was extremely high in orthogonal hybrids (ZTQ) and reciprocal hybrids (FTQ), reaching 32.53% and 39.36%, respectively, and also accounted for 14.26% in the paternal *S. davidi* (CTQ). *Delftia* and *Sphingomonas* were also important components of the hybrid foregut microbiota, with abundances exceeding 12%. In contrast, the foregut microbiota structure of the maternal *S. prenanti* (QTQ) was relatively unique, dominated mainly by *Hyphomicrobium* (9.73%) and unclassified genera under the families Beijerinckiaceae and Hyphomicrobiales, while the abundance of *Methylobacterium* was relatively low.

In the midgut, the microbial structure underwent significant succession, with *Lactococcus* beginning to occupy a core position in some groups. The midgut microbiota of the paternal *S. davidi* (CTZ) changed drastically, with an explosive growth of *Lactococcus* reaching a relative abundance of 40.92%. Orthogonal hybrids (ZTZ) showed a similar trend, with *Lactococcus* becoming the first dominant genus (19.96%), followed by *Methylobacterium* (10.22%). However, the midgut of reciprocal hybrids (FTZ) retained high levels of *Methylobacterium* (23.07%) and *Sphingomonas* (12.66%), with a relatively lower proportion of *Lactococcus* (12.37%). The maternal parent (QTZ) exhibited a relatively uniform microbial distribution, with *Cetobacterium* (9.06%), *Staphylococcus* (6.62%), and *Aeromonas* (6.14%) being major components.

In the hindgut, *Lactococcus* and *Cetobacterium* became the most dominant genera in the community. In terms of taxonomic composition, the profile of orthogonal hybrids (ZTH) was highly similar to that of the paternal parent (CTH), both being absolutely dominated by *Lactococcus*, with relative abundances reaching 40.29% and 41.27%, respectively. In contrast, the hindgut of the maternal parent (QTH) was dominated by *Cetobacterium* (26.59%), with a *Lactococcus* abundance of only 8.34%. Reciprocal hybrids (FTH) exhibited characteristics intermediate between the parents, with *Lactococcus* (21.25%) and *Cetobacterium* (11.69%) co-dominating. These results indicate that in the hindgut, the characteristics of the dominant core microbiota in orthogonal hybrids (Z) exhibited a paternal-like compositional trend.

### 3.6. Core and Unique Microbiota Analysis

**Figure 9:**
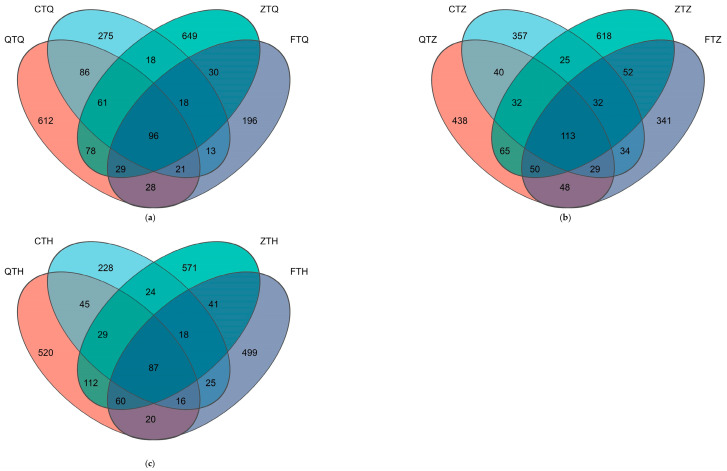
Venn diagrams illustrating the shared and unique ASVs of intestinal microbiota across different intestinal segments in parental and hybrid *Schizothorax* groups. The diagrams display the number of shared (overlapping regions) and unique (non-overlapping regions) ASVs among the four groups in the (**a**) Foregut, (**b**) Midgut, and (**c**) Hindgut. The central overlapping region represents the core microbiome shared by all groups. Abbreviations: Q: *S. prenanti*; C: *S. davidi*; Z: Orthogonal hybrid (*S. davidi* ♂ × *S. prenanti* ♀); F: Reciprocal hybrid (*S. prenanti* ♂ × *S. davidi* ♀).

Venn diagram analysis of the foregut (Figure 9a) showed that the four experimental groups shared 96 core Amplicon Sequence Variants (ASVs). Regarding the number of unique taxa, orthogonal hybrids (ZTQ) possessed the most unique ASVs (649), followed by the maternal *S. prenanti* (QTQ, 612) and paternal *S. davidi* (CTQ, 275), while reciprocal hybrids (FTQ) had the fewest (196). In terms of pairwise sharing, orthogonal hybrids (ZTQ) shared 264 ASVs with the maternal parent (QTQ) and 193 ASVs with the paternal parent (CTQ); reciprocal hybrids (FTQ) shared 174 ASVs with the maternal parent (QTQ) and 148 ASVs with the paternal parent (CTQ).

Venn diagram analysis of the midgut (Figure 9b) showed that the four groups shared 113 core ASVs. Orthogonal hybrids (ZTZ) exhibited the highest number of unique ASVs in the midgut, reaching 618, which was higher than that of the parents (QTZ: 438, CTZ: 357) and reciprocal hybrids (FTZ: 341). Regarding shared microbiota, orthogonal hybrids (ZTZ) shared 260 ASVs with the maternal parent (QTZ) and 202 ASVs with the paternal parent (CTZ); reciprocal hybrids (FTZ) shared 240 ASVs with the maternal parent (QTZ) and 208 ASVs with the paternal parent (CTZ).

Venn diagram analysis of the hindgut (Figure 9c) showed that the four groups shared 87 core ASVs. The number of unique ASVs varied greatly among groups: orthogonal hybrids (ZTH) possessed 571 unique ASVs, the maternal *S. prenanti* (QTH) had 520, reciprocal hybrids (FTH) had 499, and the paternal *S. davidi* (CTH) had the fewest (228). In ASV sharing statistics, orthogonal hybrids (ZTH) shared 288 ASVs with the maternal parent (QTH) and 158 ASVs with the paternal parent (CTH); reciprocal hybrids (FTH) shared 183 ASVs with the maternal parent (QTH) and 146 ASVs with the paternal parent (CTH).

### 3.7. Analysis of Differential Indicator Microbiota Across Intestinal Segments

**Figure 10:**
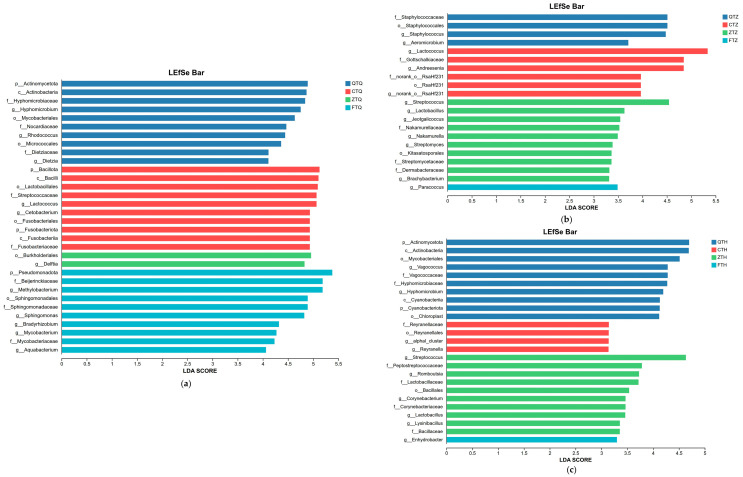
Linear discriminant analysis Effect Size (LEfSe) identifying taxonomic biomarkers of intestinal microbiota among parental and hybrid *Schizothorax* groups in different intestinal segments. The histograms display the LDA scores of differentially abundant taxa in the (**a**) Foregut, (**b**) Midgut, and (**c**) Hindgut. The length of the bar represents the logarithmic LDA score, and the color indicates the group in which the taxon is significantly enriched (Kruskal–Wallis test, *p* < 0.05). Note: To optimize visualization due to the varying number of differential taxa, the LDA score threshold was set to 4.0 for the foregut (**a**) and 2.0 for the midgut (**b**) and hindgut (**c**). Abbreviations: Q: *S. prenanti*; C: *S. davidi*; Z: Orthogonal hybrid (*S. davidi* ♂ × *S. prenanti* ♀); F: Reciprocal hybrid (*S. prenanti* ♂ × *S. davidi* ♀).

Based on LEfSe analysis (Figure 10), this study identified microbial biomarkers significantly enriched in different intestinal segments of parental and hybrid *Schizothorax*.

In the foregut (Figure 10a, LDA score > 4.0), all groups exhibited unique dominant microbiota. The maternal *S. prenanti* (Q) significantly enriched microbiota primarily including *Hyphomicrobium*, *Rhodococcus*, and *Dietzia*, members of the phylum Actinomycetota. The paternal *S. davidi* (C) was characterized by *Lactococcus* and *Cetobacterium* as indicator bacteria. In contrast, the indicator microbiota of hybrid offspring showed clear differentiation: orthogonal hybrids (Z) were significantly enriched in *Delftia* and its order Burkholderiales; reciprocal hybrids (F) were enriched in various diverse environmental genera, including *Methylobacterium*, *Sphingomonas*, *Bradyrhizobium*, *Mycobacterium*, and *Aquabacterium*.

In the midgut (Figure 10b, LDA score > 2.0), the types of indicator bacteria differed from those in the foregut. Orthogonal hybrids (Z) exhibited the highest diversity enrichment characteristics, with significant differential taxa including *Streptococcus*, *Lactobacillus*, *Jeotgalicoccus*, *Nakamurella*, *Streptomyces*, and *Brachybacterium*. The paternal *S. davidi* (C) maintained the enrichment of *Lactococcus* in the midgut, accompanied by an increase in *Andreesenia*; the maternal *S. prenanti* (Q) was enriched in *Staphylococcus* and *Aeromicrobium*; in reciprocal hybrids (F), only *Paracoccus* was detected as significantly enriched.

In the hindgut (Figure 10c, LDA score > 2.0), orthogonal hybrids (Z) were significantly enriched in taxa such as *Streptococcus*, *Lactobacillus*, *Romboutsia*, *Corynebacterium*, and *Lysinibacillus*. The maternal *S. prenanti* (Q) was enriched in *Vagococcus* and *Hyphomicrobium*; markers for the paternal *S. davidi* (C) were *Reyranella* and alphaI_cluster; reciprocal hybrids (F) were characterized by *Enhydrobacter* as the differential genus.

### 3.8. Co-Occurrence Network Analysis of Intestinal Microbiota

**Figure 11:**
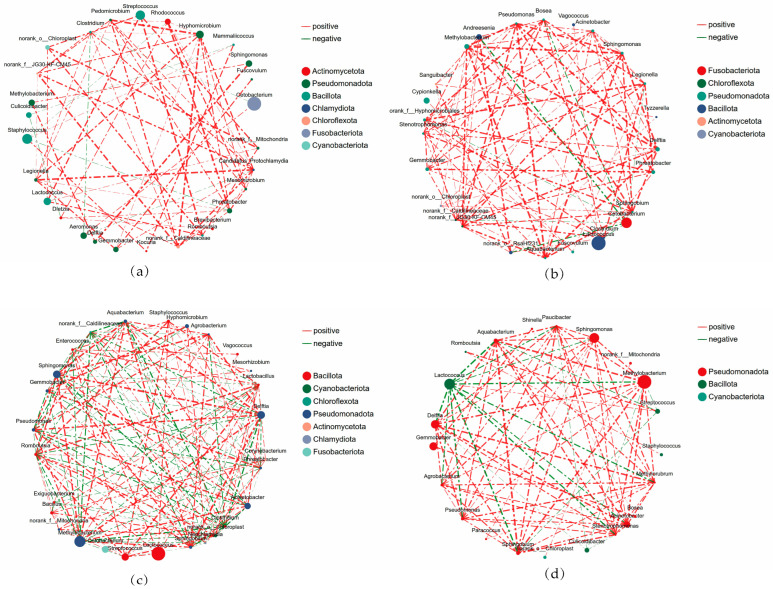
Co-occurrence network analysis of intestinal bacterial communities in parental and hybrid *Schizothorax* groups. The networks were constructed based on the top 30 abundant genera in each group using Spearman’s correlation coefficients. (**a**) Maternal *S. prenanti* (Q); (**b**) Paternal *S. davidi* (C); (**c**) Orthogonal hybrid (Z); (**d**) Reciprocal hybrid (F). Each node represents a bacterial genus; the size of the node is proportional to its degree of connectivity, and the color of the node indicates the phylum to which it belongs. Edges connecting nodes represent significant correlations (Spearman’s |*r*| > 0.6, *p* < 0.05); red edges denote positive correlations (co-occurrence), while green edges denote negative correlations (exclusion).

To elucidate the internal ecological interactions within the *Schizothorax* intestinal microbial community and the influence of hybridization on microecological patterns, this study constructed co-occurrence networks for parental and hybrid offspring based on the top 30 genera in relative abundance (Figure 11).

The intestinal microbial interaction network of *S. prenanti* (Figure 11a) consisted of 28 nodes and 66 significant edges (Spearman |*r*| > 0.6, *p* < 0.05). The network transitivity was 0.673, with 59 positive correlation edges, accounting for 89.4%. Regarding degree centrality, *Candidatus Protochlamydia* exhibited the highest connectivity (Degree = 11), followed by *Hyphomicrobium* (Degree = 10), *Pedomicrobium* (Degree = 8), and *Legionella* (Degree = 8). Significant positive correlations existed among these genera (Weight > 0.8). Seven negative correlation edges were identified in the network, accounting for 10.6%, including negative correlations between *Aeromonas* and *Candidatus Protochlamydia* (*r* = −0.63) as well as *Clostridium* (*r* = −0.66), and between *Staphylococcus* and *Phreatobacter* (*r* = −0.64).

The intestinal microbial interaction network of *S. davidi* (Figure 11b) contained 26 nodes and 91 significant edges, with a network transitivity of 0.739. *Acinetobacter* exhibited the highest degree centrality with a connectivity of 12. *Acinetobacter* showed significant positive correlations with *Gemmobacter*, *Phreatobacter*, *Legionella*, and *Sphingomonas*. The dominant genus *Cetobacterium* had a connectivity of 7, with its edges primarily composed of negative correlations, including those with *Bosea* (*r* = −0.75), *Andreesenia* (*r* = −0.86), *Legionella* (*r* = −0.60), and norank_f__Hyphomicrobiales (*r* = −0.63). The connectivity of *Lactococcus* in this network was 1.

The intestinal microbial network of orthogonal hybrids (Figure 11c) contained 28 nodes and 160 significant edges, with a network transitivity of 0.700. The number of negative correlation edges in this network was 70, accounting for 43.8%. *Romboutsia* had the highest connectivity (Degree = 19), while *Clostridium*, *Methylobacterium*, and *Aquabacterium* all had a connectivity of 17, and *Lactobacillus* and *Delftia* had a connectivity of 16. Significant positive correlations were observed among *Romboutsia*, *Lactobacillus*, *Streptococcus*, and *Clostridium*. Meanwhile, *Romboutsia* was negatively correlated with *Methylobacterium* (*r* = −0.78), *Sphingomonas* (*r* = −0.75), and *Acinetobacter* (*r* = −0.74); *Clostridium* was negatively correlated with *Delftia* (*r* = −0.86) and *Sphingomonas* (*r* = −0.84).

The intestinal microbial network of reciprocal hybrids (Figure 11d) contained 22 nodes and 83 significant edges, with a network transitivity of 0.885. *Methylobacterium* possessed the highest connectivity (Degree = 13), followed by *Acinetobacter*, *Delftia*, *Sphingobium*, *Aquabacterium*, and *Lactococcus*, all with a connectivity of 12. A tight positive correlation network was constructed among members of the phylum Pseudomonadota, represented by *Methylobacterium*, *Acinetobacter*, *Delftia*, and *Sphingobium*. The correlation coefficient between *Acinetobacter* and *Delftia* was as high as 0.92, and *Sphingobium* and *Pseudomonas* also exhibited an extremely strong synergistic relationship (*r* = 0.86).

### 3.9. Correlation Analysis Between Intestinal Microbiota and Digestive Enzyme Activities

**Figure 12:**
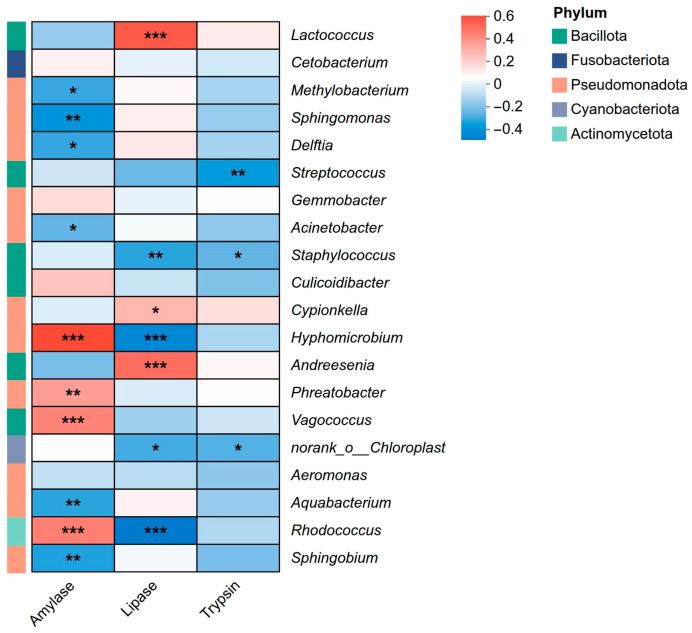
Spearman correlation heatmap between dominant bacterial genera and digestive enzyme activities in *Schizothorax* intestine. Each row represents a bacterial genus, and each column represents a digestive enzyme. The color of the heatmap indicates the Spearman correlation coefficient (r), with red indicating positive correlation and blue indicating negative correlation. Asterisks within cells denote statistical significance (* FDR-adjusted *p* < 0.05, ** FDR-adjusted *p* < 0.01, *** FDR-adjusted *p* < 0.001).

To further elucidate the potential contribution of the intestinal microbial community to host digestive function, this study conducted Spearman correlation analysis between the top 20 dominant genera in relative abundance and the activities of three key digestive enzymes (Figure 12).

The results showed that amylase activity exhibited a significant positive correlation with the characteristic dominant microbiota of *S. prenanti* (group Q). Specifically, *Hyphomicrobium* (*r* = 0.55, *p* < 0.001), *Rhodococcus* (*r* = 0.40, *p* < 0.01), and *Vagococcus* (*r* = 0.39, *p* < 0.01) all showed extremely significant positive correlations with amylase activity. Conversely, this enzyme activity showed significant negative correlations with ubiquitous environmental genera widely present in reciprocal hybrids (group F), such as *Sphingomonas* (*r* = −0.36), *Delftia* (*r* = −0.30), and *Methylobacterium* (*r* = −0.30). Lipase activity exhibited an extremely strong positive correlation (*r* = 0.51, *p* < 0.001) with *Lactococcus*, the core dominant genus of *S. davidi* (group C) and orthogonal hybrids (group Z). Additionally, *Andreesenia* (*r* = 0.45, *p* < 0.001) also showed a significant positive correlation with lipase activity. Notably, *Rhodococcus* and *Hyphomicrobium*, which were positively correlated with amylase, exhibited significant negative correlations with lipase activity here (*r* = −0.47, *r* = −0.41). In contrast, trypsin activity had weak correlations with most dominant genera, showing no strong positive correlation groups, and only exhibiting limited negative correlations with *Streptococcus* (*r* = −0.34, *p* < 0.01) and *Staphylococcus* (*r* = −0.25, *p* < 0.05).

## 4. Discussion

### 4.1. Divergence of Morphological and Physiological Functions of Digestive Organs

The morphological structure and physiological characteristics of the fish digestive tract possess high plasticity and often undergo adaptive evolution in response to feeding habits. Previous studies have well established the trophic differentiation of the parental species in sympatric environments. Wang et al. confirmed the specialized herbivorous traits of *S. prenanti* [15], while recent research on Schizothoracinae has delineated the carnivorous nutritional niche of *S. davidi* [16]. Building on this established ecological context, our study reveals the specific micro-morphological strategies they evolved to support these distinct habits. Consistent with the strategy of carnivorous fish to compensate for a shorter gut, *S. davidi* exhibited distinct microscopic characteristics by expanding its absorptive surface area. This was achieved by significantly increasing the height and width of intestinal folds. This combination of mucosal surface expansion and high lipase activity facilitates its efficient utilization of high-energy animal-based diets [8]. In contrast, *S. prenanti* has evolved a typical herbivorous strategy. Complementing its elongated intestine reported in the literature, our data shows it has evolved a significantly thickened intestinal inner circular muscle layer corresponding with the highest amylase activity. This morphological and physiological synergy not only supports vigorous intestinal peristalsis for mechanically breaking down plant cell walls but also ensures efficient hydrolysis of high carbohydrates in plant-based feeds. This aligns with the findings of Hidalgo et al., who confirmed that intestinal amylase activity in herbivorous or omnivorous fish is significantly higher than that in carnivorous fish and the enzyme profile strictly matches their feeding habits [17].

Digestive strategies exhibited divergent patterns in hybrid offspring. Interestingly, both orthogonal and reciprocal hybrids exhibited significantly higher lipase activities compared to the maternal *S. prenanti*. This result indicates that the high lipid utilization capacity of the paternal parent was effectively retained in both hybridization directions. However, the orthogonal hybrids exhibited a comprehensive digestive advantage that was absent in the reciprocal hybrids. Specifically, the orthogonal hybrids maintained significantly higher amylase activity that was comparable to the maternal parent. In contrast, the reciprocal hybrids exhibited the lowest amylase activity among all groups. Consequently, the orthogonal hybrids effectively combined the maternal capacity for carbohydrate hydrolysis with the paternal capacity for lipid digestion. The reciprocal hybrids showed a functional mismatch where high lipid digestion capability was not supported by efficient carbohydrate digestion. This physiological imbalance is often attributed to metabolic disorders caused by reduced genomic compatibility in distant hybridization [18,19]. Furthermore, no significant differences were detected in trypsin activity among any groups. This suggests that protein digestion capability is evolutionarily conserved in *Schizothorax* species to maintain basic somatic growth, consistent with findings that proteolytic activity often remains stable across distinct trophic guilds [20]. Therefore, the or-thogonal combination demonstrates a more balanced physiological potential than the re-ciprocal combination. et al.

### 4.2. Remodeling Effect of Hybridization on Alpha Diversity and Spatial Distribution of Intestinal Microbiota in Schizothorax

Host genetic background is a crucial factor shaping the structure of the intestinal microbial community. This study found that hybridization significantly altered the Alpha diversity of the *Schizothorax* intestinal microbiota and its spatial distribution pattern along the digestive tract. Notably, orthogonal hybrids exhibited significant spatial heterogeneity in the intestine, with Shannon indices in the foregut and midgut being significantly higher than those in the hindgut, whereas the spatial distribution in parental groups was relatively uniform. This result implies that in the absence of strong selective pressure, the assembly of microbial communities is largely influenced by stochastic dispersal and migration [21].

Typically, the fish foregut, as the initiation site of digestion, is susceptible to the continuous influx of transient bacteria from the aquatic environment and food [22,23]. The high diversity observed in the foregut of orthogonal hybrids in this study may be attributed to hybridization-induced changes in feeding behavior or variations in digestive tract morphology, which increase the capacity to accommodate exogenous microorganisms. Particularly when parents possess different feeding habits, hybrid offspring often exhibit a nutritional niche intermediate between the parents or even broader; such dietary heterosis has been proven to significantly reshape the assembly of the intestinal microbiota and increase microbial diversity [24]. As chyme moves towards the hindgut, drastic changes in environmental conditions, such as reduced pH, oxygen depletion, and alterations in bile acid concentration, exert strong selective effects [25]. This selective pressure eliminates a large number of maladapted transient bacteria, leading to the enrichment of core microbiota responsible for fermentation functions, thereby resulting in decreased species richness but reduced functional redundancy in the hindgut [26]. The emergence of this spatial heterogeneity implies that hybridization may reshape the micro-niche of symbiotic microorganisms by altering the physicochemical properties and immune factor secretion of the host intestine [27].

### 4.3. Genetic Imprinting and Colonization Mechanisms of Intestinal Microbiota

Combining Beta diversity and Venn diagram analysis, this study reveals the complexity of intestinal microbiota assembly in hybrid offspring. In the midgut, PCoA analysis showed that the community structure of orthogonal hybrids was closer to their paternal parent (*S. davidi*), mainly reflecting the similarity of high-abundance dominant microbiota; however, Venn diagram analysis indicated that orthogonal hybrids shared a greater number of ASVs with their maternal parent (*S. prenanti*). This phenomenon of structural bias towards the paternal parent and compositional bias towards the maternal parent aligns with findings from genome-wide association studies in other vertebrates, indicating that the influence of host genotype on the microbiome is locus-specific and taxa-specific [28].

The colonization of dominant microbiota may be primarily influenced by the intestinal physiological environment regulated by the host nuclear genome. The host’s major histocompatibility complex (MHC) genes can regulate the colonization abundance of dominant symbiotic bacteria through specific recognition mechanisms. Orthogonal hybrids may have inherited more competitively advantageous immune genes or mucin genes from the paternal parent, thereby exhibiting paternal dominance in the assembly of dominant microbiota [29]. In contrast, the colonization of low-abundance or rare microbiota may originate more from maternal environmental exposure during early life history stages, thus retaining the imprint of the maternal species pool [30]. However, given that the fish were reared in the same recirculating system from the fertilized egg stage to adulthood (2 years), the persistent microbial differences among groups likely reflect the stable selection of host genetic background rather than transient early environmental effects. Furthermore, the structural convergence of hindgut microbiota observed across all groups suggests that, although genetic background plays a dominant role in the foregut and midgut, the strong functional selective pressure in the hindgut may override genetic factors, forcing hosts with different genotypes to evolve similar core fermentative microbiota to maintain basic energy metabolism. This is consistent with the core microbiota phenomenon observed in zebrafish [31,32].

### 4.4. Spatial Succession of Core Microbiota and Diet-Driven Compositional Differences

This study shows that the *Schizothorax* intestinal microbial community exhibits significant spatial succession characteristics along the digestive tract. At the phylum level, the foregut of all groups was absolutely dominated by Pseudomonadota, especially in hybrid offspring where the abundance exceeded 90%. This result is consistent with studies on *Schizothorax nukiangensis* [33], *Ctenopharyngodon idellus* [34], and *Cyprinus carpio* [35], all of which identified Pseudomonadota as the major dominant taxon in the freshwater fish intestinal microbiota. As chyme moves to the hindgut, the intestinal microenvironment changes, and the abundances of Bacillota and Fusobacteriota increase, replacing Pseudomonadota as the dominant taxa in the midgut and hindgut. This phylum-level succession reflects the strong screening effect of specific physicochemical environments, such as anaerobiosis within the host intestine, on symbiotic microbiota with fermentation functions, promoting the enrichment of core microbiota in the hindgut to perform metabolic functions [36].

Genus-level analysis further reveals spatial and inter-group variations in the intesti-nal microbiota. The foregut of both hybrid groups was enriched with environmentally bacteria such as *Methylobacterium* and *Delftia*, sharing certain taxonomic similarities with the paternal *S. davidi*. As digestion proceeds to the midgut, orthogonal hybrids exhibited an early succession characterized by a rapid emergence of *Lactococcus*, a crucial lactic acid bacterium typically associated with protein and lipid rich diets [37], showing a compositional shift that partially reflects the paternal profile, whereas reciprocal hybrids retained a high proportion of foregut-associated *Methylobacterium*. In the hindgut, despite the overall statistical convergence of the microbial community across groups, descriptive observations of the taxonomic profiles still revealed distinct functional strategies: the herbivorous maternal *S. prenanti* maintained a notable proportion of Ceto-bacterium, an autochthonous bacterium that excels in fermenting carbohydrates [38], while the paternal *S. davidi* and orthogonal hybrids both exhibited high relative abundances of *Lactococcus* (over 40%). These observations raise the possibility that orthogonal hybrids might potentially deploy a microecological strategy sharing features with the paternal parent, which could be advantageous for extracting energy from high-nutritional-value diets. This hypothesis is further supported by the functional prediction analysis based on PICRUSt2 (Figure S2).

LEfSe differential analysis further revealed the functional significance behind this community succession. Orthogonal hybrids not only mirrored the taxonomic profile of the paternal dominant bacteria but also specifically enriched lactic acid bacteria represented by *Lactobacillus* and *Streptococcus*, as well as *Cetobacterium*, throughout the entire intestine. Among them, *Cetobacterium*, as a core autochthonous bacterium of freshwater fish intestines, has been confirmed to synthesize vitamin B12 and efficiently ferment carbohydrates to produce acetate [39]. This foundational understanding of microbiome-mediated interactions is further supported by recent studies demonstrating the crucial impact of gut microbiota on host physiological resilience [40]. Acetate is not only a direct energy substrate for host peripheral tissues but also promotes host lipid metabolism and protein synthesis by regulating insulin sensitivity [41]. More importantly, the *Lactobacillus* significantly enriched in orthogonal hybrids is a recognized probiotic. *Lactobacillus* species in freshwater fish intestines not only demonstrate strong antagonistic activity against common aquatic pathogens like *Aeromonas* by secreting organic acids and bacteriocins to inhibit pathogen growth but have also been proven to possess the potential to secrete extracellular amylase, lipase, and protease [37,42]. The diverse probiotic potentials of such beneficial bacteria in modulating intestinal health and defending against pathogens have been extensively corroborated by recent advances in the field [43]. Additionally, orthogonal hybrids specifically enriched *Delftia* in the foregut, a genus with the potential to degrade complex organic pollutants, which may enhance the host’s tolerance to anti-nutritional factors in feed [44]. In contrast, reciprocal hybrids mainly enriched *Methylobacterium* and *Sphingomonas*. These microbiota are typically widespread in aquatic environments and on algal biofilm surfaces [45]; their excessive enrichment in reciprocal hybrids may imply a weaker capability of their intestine to resist exogenous bacterial colonization, making them more susceptible to invasion by environmental microorganisms, or reflect a feeding habit more inclined towards filtering algae. Importantly, the rearing water microbiome was not sequenced, warranting future investigations to definitively trace the origin of these transient taxa.

### 4.5. Ecological Interaction Mechanisms Revealed by Co-Occurrence Network Analysis

The function of a microbial community depends not only on species abundance but is also profoundly influenced by interaction patterns among species. Through co-occurrence network analysis, this study found that hybridization markedly influenced the network of orthogonal hybrids had the highest number of edges and the highest node degree. Such a highly complex network structure typically signifies highly interconnected micro-ecosystem, which theoretically provides more diverse path-ways for microbial metabolic cross-feeding and interactions [46]. In contrast, the microbial network of *S. prenanti* had a positive correlation edge proportion as high as 89.4%, exhibiting an interaction pattern dominated by mutual cooperation. Although this structure facilitates the synergistic utilization of nutrients by the microbiota, networks overly reliant on positive correlations are theoretically more sensitive to environmental perturbations, as damage to core nodes can easily trigger cascading reactions leading to community structure instability [47].

Notably, prominent negative correlation interactions were observed in *S. davidi* and hybrid offspring, which in ecology typically corresponds to interspecific competition or niche differentiation. In *S. davidi*, the core dominant bacterium *Cetobacterium* showed strong negative correlations with various bacteria of environmental origin. Previous studies indicate that *Cetobacterium* is an autochthonous bacterium in freshwater fish intestines, capable of fermenting carbohydrates to produce acetate, thereby lowering intestinal pH [39]. The negative associations observed in this study reflect that autochthonous bacteria in the *S. davidi* intestine may inhibit the colonization of non-acid-tolerant exogenous bacteria by altering the microenvironment, thus establishing a natural biological barrier.

In orthogonal hybrids, this competition-based defense characteristic was even more pronounced. The proportion of negative correlation edges in this group’s network reached 43.8%, forming two functionally distinct microbial cliques: one centered on fermentative bacteria like *Romboutsia* and *Lactobacillus*, and the other represented by environmentally adaptable bacteria like *Methylobacterium* and *Acinetobacter*. Data analysis showed a strong negative correlation between these two groups of bacteria. Studies in cyprinid fishes have confirmed that an increase in *Romboutsia* abundance is usually accompanied by elevated intestinal acetate concentrations, which can not only provide direct energy for the host intestinal epithelium but also maintain intestinal barrier function by lowering the microenvironmental pH [48]. In this study, the potential association between *Romboutsia* and lipase activity may imply that it indirectly supports the host’s inferred capacity for lipid digestion by improving the intestinal energy metabolism status. The retention of a large number of such fermentative bacteria in the intestine of orthogonal hybrids, forming a competitive exclusion against environmental bacteria, suggests that their intestinal environment may have exerted directional selection on microorganisms, inhibiting the excessive proliferation of environmental bacteria. Given the compositional nature of 16S data, these Spearman-based co-occurrence networks should be strictly interpreted as preliminary patterns rather than definitive ecological stability.

In comparison, the network of reciprocal hybrids showed the highest transitivity, indicating highly clustered characteristics within the microbiota, but its core positions featured environmental taxa such as *Methylobacterium*. Given its known environmental origins, its prominent topological position within these specific intestinal networks must be interpreted with caution. Rather than representing an established, active resident (autochthonous) mutualism, this high connectivity may reflect a coordinated, group-specific transient accumulation from the rearing water, permitted by the divergent physiological microenvironment of the reciprocal hybrid’s foregut. Although *Lactococcus* in this group also exhibited antagonistic effects against environmental bacteria, it lacked the support of abundant and highly connected fermentative microbial groups seen in orthogonal hybrids. This indicates that different directions of hybridization lead to different microbiota assembly strategies: the microbial network of orthogonal hybrids is dominated by fermentative functional bacteria and exerts repulsion against exogenous bacteria, whereas the network of reciprocal hybrids appears more susceptible to the transient influx of environmental microorganisms. We found that orthogonal hybrids, by constructing a network structure with high complexity and strong competitiveness, reinforced the inhibitory effect of core beneficial bacteria on environmental bacteria. This optimized microecological interaction pattern may provide an important microbiological basis for the inferred physiological potential they exhibit in aquaculture environments.

### 4.6. Synergistic and Antagonistic Relationships Between Intestinal Microbiota and Digestive Enzyme Activities

The correlation analysis in this study revealed specific correspondences between dominant bacterial genera and host digestive enzyme activities. As a representative herbivore, the high intestinal amylase activity in *S. prenanti* showed extremely significant positive correlations with *Hyphomicrobium* and *Rhodococcus*. *Rhodococcus* sp. (MTCC 9508) isolated from fish intestines by Khan et al. was not only confirmed as a high-efficiency phytase producer but was also found to possess the ability to secrete extracellular amylase [49]. *Hyphomicrobium* species can provide carbon sources for the organism and are key players in anaerobic systems [50]. These genera typically possess the ability to degrade cellulose and complex polysaccharides; their colonization in the intestine may assist in enhancing the host’s carbohydrate utilization efficiency by converting indigestible plant polysaccharides into short-chain fatty acids available to the host [51]. In sharp contrast, *S. davidi* and orthogonal hybrids exhibited clear characteristics of elevated lipase activity, with their lipase activity showing an extremely strong positive correlation with *Lactococcus*. *Lactococcus* is a recognized probiotic in the intestine of aquatic animals, capable of producing extracellular lipase; its high-abundance colonization in the intestine of orthogonal hybrids is hypothesized to be associated with the host’s overall lipolytic profile, though its precise functional contribution requires future metagenomic validation [38]. Interestingly, *Rhodococcus* and *Hyphomicrobium*, which were positively correlated with amylase, exhibited significant negative correlations with lipase activity here. This phenomenon indicates that orthogonal hybrids exhibit an intestinal microecology characterized by with potential lipase-associated capabilities consistent with the paternal parent, achieving paternal inheritance of an enhanced intestinal lipolytic capacity.

The relationship between microbiota and enzyme activity in reciprocal hybrids insights into the potential microecological factors associated with their poor digestive function. Environmentally derived bacteria such as *Sphingomonas*, *Delftia*, and *Methylobacterium*, which are widespread in reciprocal hybrids, were significantly negatively correlated with host amylase activity. This negative association suggests that the dominant environmental microbiota in the intestine of reciprocal hybrids lacks a synergistic relationship with host enzymatic secretion. Furthermore, it could theoretically reflect a competitive dynamic for nutritional substrates within the intestine, potentially contributing to a mismatch between microecological functions and host physio-logical needs [19]. Furthermore, trypsin activity had weak correlations with most dominant genera and even showed some negative correlation with *Streptococcus*. This result is consistent with previous findings in other teleosts, namely that protein digestion relies mainly on trypsin secreted by the host itself, while the direct contribution of intestinal microbiota to protein digestion is relatively limited. Excessive proliferation of certain proteolytic bacteria might even produce toxic metabolic byproducts, thereby exerting adverse effects on host health [23]. Furthermore, these microbe-enzyme correlations are likely driven by macroscopic genotypic divergence across groups rather than implying direct causal links. Without direct metabolomic evidence, the actual metabolic outputs of these specific taxa remain predictive and require future empirical validation.

## 5. Conclusions

Through multidimensional comparative analysis, this study elucidates the impact of interspecific hybridization in the genus *Schizothorax* on the intestinal characteristics and microecological assembly of offspring. The main conclusions are as follows:

(1) *S. prenanti* and *S. davidi* exhibited distinct phenotypic features that closely corresponded to their feeding habits. *S. prenanti* possessed developed intestinal inner circular muscle layers, high amylase activity, and a *Cetobacterium*-rich microbiota, reflecting its habit of scraping plant-based food. In contrast, *S. davidi* exhibited traits associated with its consumption of animal-based food, including developed intestinal mucosal folds, high lipase activity, and a *Lactococcus*-rich microbiota.
(2) The orthogonal hybrids (Z) exhibited significant paternal bias in digestive physiology and microbial community assembly. They successfully inherited the high lipase activity and the capacity for *Lactococcus* enrichment from the paternal *S. davidi*, thereby demonstrating an inferred physiological potential for enhanced lipid digestion. Furthermore, the orthogonal hybrids constructed the most complex microbial interaction network, displaying highly intricate co-occurrence patterns.
(3) The reciprocal hybrids (F) showed a marked discordance between intestinal morphology and physiological function. Although their hindgut fold height was significantly higher than that of other groups, both amylase and lipase activities remained at low levels; this morphological advantage failed to translate into an improvement in actual digestive capacity. Simultaneously, the core microbiota of reciprocal hybrids was occupied by environmental bacteria such as *Methylobacterium*, lacking the support of stable functional symbiotic groups.
(4) Our findings suggest a putative spatially dependent assembly pattern: the structural divergence of the microbiome in the anterior segments (foregut and midgut) aligns strongly with host genetic background, whereas the hindgut microbiota exhibits structural convergence under the long-term administration of a standardized diet. This indicates that the orthogonal hybrid (with *S. davidi* as the paternal parent) possesses specific digestive physiological profiles that warrant further investigation under artificial farming environments.

In summary, this study provides baseline profiles of the divergent intestinal lipolytic capacities and distinct microecological assembly patterns driven by reciprocal hybridization. Based on the findings of this study, future work should further integrate multi-omics technologies to deeply elucidate the molecular regulatory mechanisms underlying the elevated lipase activity and complex microecological networks in orthogonal hybrids. Meanwhile, functional verification of core microbiota enriched in orthogonal hybrids, such as *Lactococcus* and *Romboutsia*, is recommended to confirm their specific contributions to host digestion. Importantly, these intestinal and microecological profiles represent baseline traits. Future on-farm performance validations, including systematic evaluations of growth rates, feed conversion ratios, and long-term survival, are strictly required before these inferred potentials can be translated into applied aquaculture recommendations.

## Figures and Tables

**Table 1:** Morphometric characteristics of the intestinal tract in parental and hybrid *Schizothorax* groups.

Part	Metric (μm)	Q	C	Z	F	*p*-Value
Anterior Intestine	FH	522.26 ± 90.59 ^c^	749.49 ± 116.46 ^a^	502.51 ± 119.60 ^c^	603.69 ± 177.80 ^b^	<0.001
	FW	119.09 ± 23.99 ^b^	138.93 ± 27.08 ^a^	120.72 ± 29.88 ^b^	110.83 ± 31.74 ^b^	0.001
	ICM	114.79 ± 25.40 ^a^	77.65 ± 25.12 ^b^	92.56 ± 28.15 ^b^	80.78 ± 24.50 ^b^	<0.001
	OLM	57.74 ± 11.06 ^a^	44.71 ± 15.06 ^b^	59.14 ± 10.32 ^a^	52.77 ± 13.77 ^ab^	<0.001
Mid-intestine	FH	312.90 ± 72.62 ^b^	384.88 ± 95.90 ^a^	297.61 ± 44.83 ^b^	394.79 ± 78.46 ^a^	<0.001
	FW	87.80 ± 18.82 ^b^	99.96 ± 17.01 ^a^	89.09 ± 12.77 ^b^	87.28 ± 17.51 ^b^	0.002
	ICM	63.20 ± 10.03 ^a^	36.79 ± 6.54 ^c^	49.98 ± 9.92 ^b^	47.50 ± 10.62 ^b^	<0.001
	OLM	38.22 ± 7.65 ^a^	28.37 ± 6.62 ^b^	42.25 ± 10.67 ^a^	37.03 ± 9.24 ^a^	<0.001
Posterior Intestine	FH	286.44 ± 64.18 ^b^	441.25 ± 97.16 ^a^	283.57 ± 43.62 ^b^	412.24 ± 82.06 ^a^	0.002
	FW	91.58 ± 13.05 ^a^	101.01 ± 20.61 ^a^	93.85 ± 13.72 ^a^	97.52 ± 20.36 ^a^	0.113
	ICM	57.85 ± 11.82 ^ab^	45.50 ± 7.52 ^b^	47.92 ± 8.90 ^ab^	63.24 ± 36.26 ^a^	0.018
	OLM	40.98 ± 7.43 ^a^	34.46 ± 10.92 ^a^	34.86 ± 5.87 ^a^	41.52 ± 13.92 ^a^	0.019

Note: Data are presented as mean ± SD (*n* = 5). Different superscript lowercase letters within the same row indicate significant differences among groups (*p* < 0.05, Tukey’s HSD test). The *p*-value column represents the overall significance from the One-way ANOVA. Abbreviations: Q: *Schizothorax* prenanti; C: *S. davidi*; Z: Orthogonal hybrid (*S. davidi* ♂ × *S. prenanti* ♀); F: Reciprocal hybrid (*S. prenanti* ♂ × *S. davidi* ♀). FH: fold height; FW: fold width; ICM: inner circular muscle thickness; OLM: outer longitudinal muscle thickness.

## Data Availability

The original contributions presented in this study are included in the article. Further inquiries can be directed to the corresponding authors.

## Supplementary Materials

The following supporting information can be downloaded at: https://www.mdpi.com/article/10.3390/ani16060895/s1, Figure S1. Schematic illustration of the morphometric measurement methodology in the fish intestine. Figure S2. Predicted functional profiles of the intestinal microbiota based on PICRUSt2 analysis. Table S1. Global PERMANOVA (Adonis) and homogeneity of multivariate dispersions (betadisper) results based on Bray-Curtis dissimilarity matrices for intestinal microbiota across different segments.
