# Supplementary material for: Reciprocal Hybridization Between Herbivorous and Carnivorous Sub-Cold-Water Fish Reveals Divergent Intestinal Characteristics and Microbiome Assembly
Source: Animals (Basel). 2026 Mar 12;16(6):895. doi: 10.3390/ani16060895 (PMC13023282; doi:10.3390/ani16060895)
Supplement: Supplementary file 1 [file animals-16-00895-s001.zip › animals-4158657-supplementary.pdf]

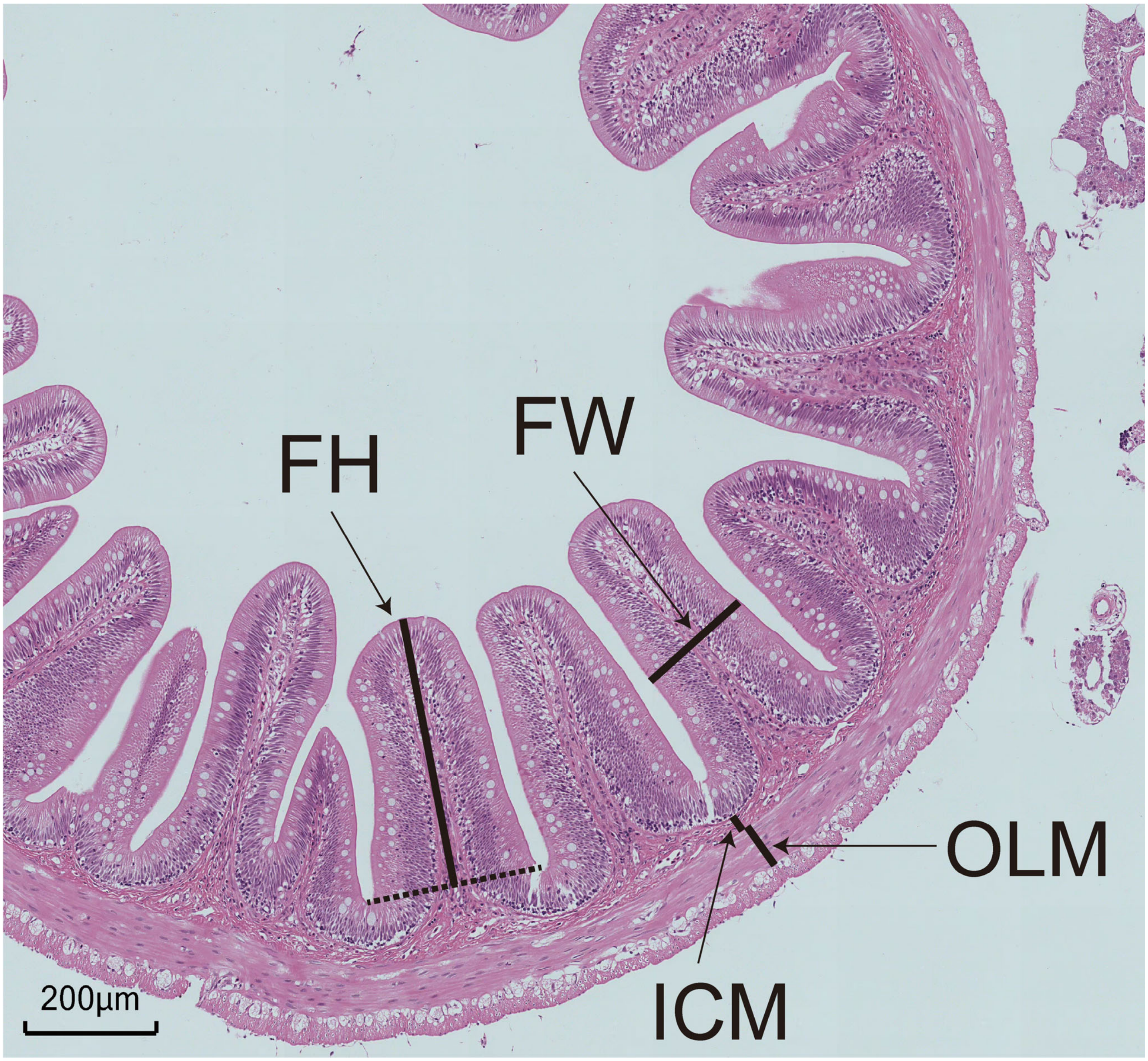

Figure S1. Schematic illustration of the morphometric measurement methodology in the fish intestine. A representative high-resolution micrograph of a transverse intestinal section (H&E staining) is presented to explicitly demonstrate the exact anatomical locations and methods used for acquiring the four key morphological parameters. All measured parameters are indicated by solid black lines, while the auxiliary reference baseline (connecting the bases of the mucosal fold) is indicated by a dashed black line. FH (fold height): the solid line distance extending from the midpoint of the auxiliary dashed line to the tip of the fold; FW (fold width): the solid line distance horizontally across the middle of the fold; ICM (inner circular muscle thickness): the solid line distance between the inner and outer boundaries of the inner circular muscle layer; OLM (outer longitudinal muscle thickness): the solid line distance between the inner and outer boundaries of the outer longitudinal muscle layer. Scale bar = 200 μm.

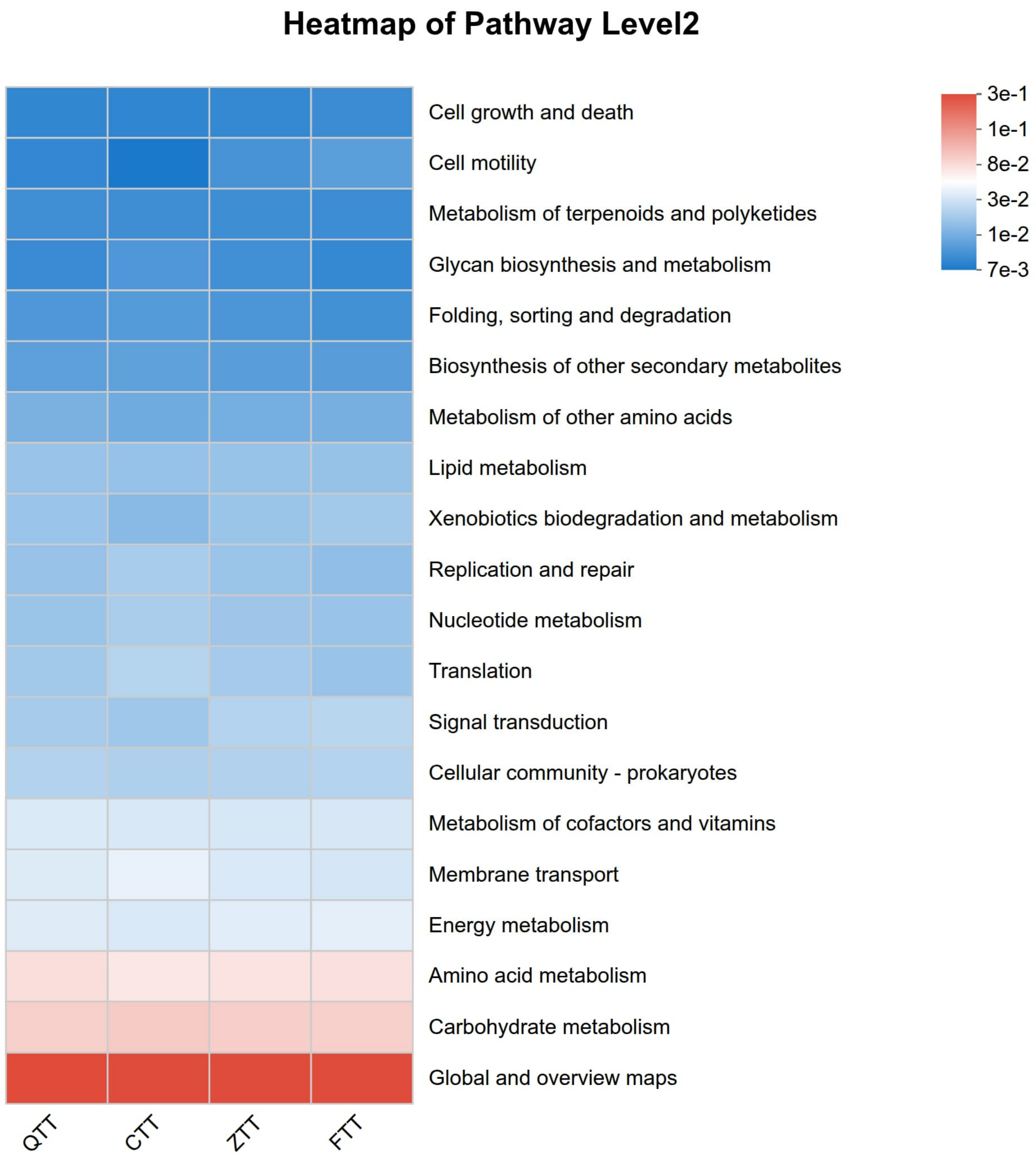

Figure S2. Predicted functional profiles of the intestinal microbiota based on PICRUSt2 analysis. The heatmap illustrates the relative abundances (Z-score normalized) of the top 20 enriched KEGG pathways at Level 2 across the four experimental groups. The color gradient from blue to red represents the relative abundance from low to high.

Table S1. Global PERMANOVA (Adonis) and homogeneity of multivariate dispersions (betadisper) results based on Bray-Curtis dissimilarity matrices for intestinal microbiota across different segments.

Intestinal Segment	Comparison (Groups)	Adonis R^2	Pseudo-F	Adonis *P*-value	Dispersion (*P*-value)
Foregut	Global (Q vs. C vs. Z vs. F)	0.299	2.838	0.003*	0.363
Midgut	Global (Q vs. C vs. Z vs. F)	0.236	2.059	0.008*	0.758
Hindgut	Global (Q vs. C vs. Z vs. F)	0.163	1.303	0.168	0.320
